# Comparative analysis of full-length 16s ribosomal RNA gene sequencing in human oropharyngeal swabs using primer sets with different degrees of degeneracy

**DOI:** 10.3389/fcimb.2025.1658615

**Published:** 2025-11-17

**Authors:** Christian Waechter, Janna-Nele Wilkens, Leon Fehse, Dominik Heider, Kiarash Sassani, Georgios Chatzis, Sebastian Weyand, Sabine Pankuweit, Ulrich Luesebrink, Muhidien Soufi, Jochen Pöling, Thomas Braun, Felix Ausbuettel, Volker Ruppert

**Affiliations:** 1 Faculty of Medicine, Philipps University Marburg, Marburg, Germany; 2 Clinic for Cardiology III , University Hospital Münster, Münster, Germany; 3 Department 1, Max Planck Institute for Heart and Lung Research, Bad Nauheim, Germany; 4 Department of Cardiology, University Hospital Marburg, Philipps University Marburg, Marburg, Germany; 5 Institute of Medical Informatics , University of Münster, Münster, Germany; 6 Department of Cardiology, University Hospital Greifswald, University Greifswald, Greifswald, Germany; 7 Department of Cardiology, Ostalb-Clinic, University of Ulm, Aalen, Germany; 8 Center for Undiagnosed and Rare Diseases, University Hospital Marburg, Philipps University Marburg, Marburg, Germany

**Keywords:** 16S rRNA, oral microbiome, human oropharyngeal microbiome, next-generation sequencing (NGS), nanopore sequencing, Oxford Nanopore Technologies (ONT), MinION Mk1C

## Abstract

**Background:**

Full-length 16S rRNA gene sequencing using nanopore technology has become increasingly relevant for profiling complex microbial communities, including the human oral microbiome. Primer selection plays a critical role in amplification bias and taxonomic resolution, yet remains insufficiently investigated for oropharyngeal samples.

**Methods:**

We conducted a comparative analysis of two primer sets with differing degrees of degeneracy – Oxford Nanopores (ONT) standard 27F primer (27F-I) and a more degenerate variant (27F-II) – for full-length 16S rRNA gene sequencing of 80 human oropharyngeal swab samples using ONTs MinION Mk1C. Alpha diversity and taxonomic profiles were statistically compared between primer sets and benchmarked against a large-scale salivary microbiome dataset (n=1,989) from healthy individuals.

**Results:**

Primer choice significantly impacted microbial community composition and diversity. The more degenerate primer set 27F-II yielded significantly higher alpha diversity (Shannon index: 2.684 vs. 1.850; p < 0.001) and detected a broader range of taxa across all phyla. The taxonomic profiles generated with 27F-II strongly correlated with the reference dataset (Pearson’s r = 0.86, p < 0.0001), whereas profiles generated with 27F-I showed weak correlation (r = 0.49, p = 0.06). 27F-I overrepresented Proteobacteria and underrepresented key genera such as Prevotella, Faecalibacterium, and Porphyromonas.

**Conclusion:**

Our findings demonstrate that primer degeneracy has a substantial effect on taxonomic resolution and biodiversity estimates in oropharyngeal 16S rRNA gene sequencing. The more degenerate 27F-II primer set seams to more faithfully captures the complexity of the human oropharyngeal microbiome and aligns more closely with population-level reference data. These results underscore the importance of careful primer selection and support the adoption of degenerate primers as a methodological standard in nanopore-based oral microbiome research.

## Introduction

The human microbiome, comprising diverse and complex microbial communities, plays a crucial role in health and disease (Aggarwal et al., 2023). Among these, the oral and oropharyngeal microbiome have garnered significant interest due to the growing evidence of its role beyond general oral health, such as the involvement in respiratory infections and even systemic diseases (Bao et al., 2020; Lee et al., 2021; Peng et al., 2022). The oropharynx therefore serves as a critical interface between the upper aerodigestive tract and the external environment, making it a relevant diagnostic and research target. Compared to the gut microbiome, the oral microbiome is relatively underexplored, particularly in large-scale sequencing studies. It also differs in composition, pH, host immune interaction, and exposure to environmental factors (Huttenhower et al., 2012; Ding and Schloss, 2014). Moreover, age plays an important role: several studies have shown that the oral microbiome evolves significantly from infancy to adulthood, both in terms of taxonomic composition and stability (Sampaio-Maia and Monteiro-Silva, 2014; Burcham et al., 2020; Kageyama and Takeshita, 2024). These differences are particularly relevant when interpreting population-level data sets or making comparative references. Careful differentiation between pediatric, adolescent, and adult populations is therefore necessary. Moreover, the specific niche within the upper aerodigestive tract plays a decisive role in microbiome composition and taxonomic representation, and different anatomical sites show relevant biological differences. The oropharynx and nasopharynx, while spatially adjacent, differ substantially in epithelial lining, microbial density, immune surveillance, and exposure to environmental factors such as food, saliva, and inhaled particles (Piters et al., 2020). The oropharynx harbors a more diverse and metabolically active microbiota, with higher bacterial biomass and greater ecological connectivity to both the oral and gastrointestinal compartments (Lemon et al., 2010; Charlson et al., 2011; Huttenhower et al., 2012). Beyond these biological aspects, there are also practical advantages to studying the oropharyngeal microbiome: sampling is less invasive and more acceptable in both clinical and non-clinical settings, which facilitates routine implementation. The higher bacterial biomass further enhances the robustness and consistency of 16S rRNA gene amplification.

The rapid expansion of microbiome research has been largely driven by next-generation sequencing (NGS) technologies, which enable comprehensive, high-throughput analysis of complex microbial communities at increasingly affordable cost and turnaround times (Malla et al., 2019). Depending on read length and chemistry, sequencing platforms can broadly be categorized into short-read and long-read technologies. Short-read sequencing, most notably Illumina’s MiSeq^®^ platform (2 × 250–300 base pair), has become the most widely used approach in large-scale microbiome studies due to its high basecalling accuracy and established pipelines (Huttenhower et al., 2012; McDonalda et al., 2018; Ravi et al., 2018). However, its limited read length typically restricts analyses to partial hypervariable regions of the 16S rRNA gene—most commonly the V3–V4 or V4 region—constraining taxonomic classification primarily to the genus level and complicating comparisons across studies that target different regions (Klindworth et al., 2013; Kim et al., 2024). Moreover, species-level resolution is rarely achieved without additional genomic or functional information. Third-generation sequencing technologies such as Oxford Nanopore Technologies (ONT) overcome this limitation by generating substantially longer reads - up to 15 kilobases - enabling full-length 16S rRNA gene sequencing and improving phylogenetic resolution (Deissová et al., 2023). This is particularly advantageous for profiling complex microbial ecosystems and distinguishing closely related species. Although ONT sequencing was initially hindered by high error rates of approximately 6%, continuous improvements in flow cell design (e. g., R10.4.1), sequencing chemistry (e. g., Q20+ kits), and basecalling algorithms have markedly improved accuracy, now achieving modal read accuracies below 1% error (Kim et al., 2024). In clinical and diagnostic microbiology, ONT platforms offer additional benefits: they are compact, scalable, and enable real-time sequencing and analysis. This makes them attractive for point-of-care applications, outbreak investigations, and settings with limited laboratory infrastructure. However, challenges remain in standardization, bioinformatics pipelines, and benchmarking against short-read or whole-genome sequencing (WGS) approaches, which are still considered the gold standard for strain-level characterization and resistance profiling.

A critical source of variability in 16S rRNA gene-based microbiome profiling is the selection of primer pairs used for PCR amplification. Even minor mismatches between primer sequences and target regions - particularly in evolutionarily conserved but polymorphic regions - can introduce substantial amplification bias, leading to the preferential enrichment of certain taxa while underrepresenting others (Klindworth et al., 2013). This bias not only affects measures of alpha and beta diversity, but can also distort downstream taxonomic assignments, especially when comparing data across studies using different primer sets or targeting different regions of the gene (Deissová et al., 2023).

To address this issue, degenerate primers have been developed that incorporate nucleotide ambiguity codes at variable positions, thereby increasing coverage across a broader range of bacterial taxa. While this strategy can improve amplification inclusivity and reduce taxonomic dropout, it may also introduce challenges such as reduced amplification efficiency, increased non-specific binding, and the need for optimized PCR conditions (Frank et al., 2008).

In our previous study on human fecal samples, we systematically compared ONT’s standard 27F primer with a more degenerate variant and demonstrated that the latter resulted in significantly higher alpha diversity and a more balanced phylum-level distribution, with reduced overrepresentation of Firmicutes and Proteobacteria (Waechter et al., 2023).However, the extent to which these findings apply to other anatomical sites remains uncertain, as microbial composition, DNA extraction yield, and sequence conservation can vary widely between niches such as the gut, skin, and oral cavity (Huttenhower et al., 2012). The present study therefore extends our previous work to the oropharyngeal microbiome, a distinct and clinically relevant niche characterized by high microbial diversity and diagnostic potential. By systematically comparing primer sets in this anatomical context, our study contributes to the growing body of evidence on the influence of primer design in microbiome profiling and offer practical guidance for future studies of the oral and respiratory tract using long-read sequencing technologies.

## Materials and methods

### Sample collection and DNA extraction

Oropharyngeal swabs were collected from German donors with no history of acute systemic or oral inflammation. To ensure systematic sampling, the swabs were first applied to the teeth, tongue, and buccal mucosa before being inserted into the pharynx. Sterile swabs were used for collection and immediately transferred into tubes containing DNA/RNA shielding buffer (#R1160, Zymo Research, Irvine, CA, USA). After collection, samples were stored at room temperature and processed within three days to preserve nucleic acid integrity. Nucleic acid extraction was carried out following established protocols, ensuring purity and concentration assessment (Waechter et al., 2023). Specifically, the Quick-DNA^©^ HMW MagBead kit (#D6060, Zymo Research) was used for DNA extraction, adhering to the manufacturer’s guidelines. DNA purity and concentration were measured using a NanoDrop^©^ spectrophotometer (ThermoFisher Scientific, Waltham, MA, USA) and a Quantus^©^ Fluorometer (Promega, Madison, WI, USA). The extracted DNA was subsequently stored at -20°C for future use.

### PCR amplification and nanopore 16S rRNA gene sequencing

As previously described, two sequencing libraries were prepared from the extracted DNA, each utilizing a different primer set (Waechter et al., 2023): For the first library (referred to as the 27F-I library), 50 ng of whole genomic DNA was amplified using the 16S barcoding kit, which includes the 16S rDNA primers 27F (5’- AGAGTTTGATC**M**TGGCTCAG -3’) and 1492R (5’- CGGTTACCTTGTTACGACTT -3’), based on Escherichia coli rRNA numbering (SQK-RAB204, Oxford Nanopore Technologies, Oxford, UK). The amplification process followed the manufacturer’s protocol.

The second library (27F-II library) was generated using an alternative primer set with a higher degree of degeneracy. The first PCR amplification was performed on 50 ng of genomic DNA using the 16S rDNA primers S-D-Bact-0008-c-S-20 and S-D-Bact-1492-a-A-22 ( (Sampaio-Maia and Monteiro-Silva, 2014; Deissová et al., 2023)). These primers contained anchor sequences: 5′-TTTCTGTTGGTGCTGATATTGCAGRGTT**Y**GAT**YM**TGGCTCAG-3′ (forward) and 5′-ACTTGCCTGTCGCTCTATCTTCCGG**Y**TACCTTGTTACGACTT-3′ (reverse), followed by barcode addition through a second PCR step. The procedure followed the ONT protocol for “Ligation sequencing amplicons - PCR barcoding (SQK-LSK110 with EXP-PBC096)” (protocol available at https://nanoporetech.com/document/pcr-barcoding-96-amplicons-sqk-lsk110). The PCR protocols are published elsewhere (Waechter et al., 2023). In brief:

1. Preparation 16s-PCR: 50 ng DNA in 11.5 µl nuclease-free water, 0.5 µl Primer 27F-II, 0.5 µl Primer1492R-II, 12.5 µl LongAMP^®^ Taq 2x Master Mix (New England Biolabs, Ipswich, MA, USA). Cycle program: 1 min 95°C; 25 cycles 20 sec 95°C, 30 sec 51°C, 2 min 65°C and a 5 min final elongation at 65°C.
2. Preparation barcoding-PCR: 100 fmol 16S-PCR amplicons in 12.0 µl nuclease-free water, 0.5 µl barcode primer, 12.5 µl LongAMP^®^ Taq 2x Master Mix. Cycle program: 1 min 95°C; 15 cycles 20 sec 95°C, 30 sec 62°C, 2 min 65°C and a 5 min final elongation at 65°C.

Following barcoding-PCR, the DNA content of each amplicon was determined using Quantus™ Fluorometer and adjusted to an equal amount. The amplicons were pooled, and 1000 ng were used for library preparation. The library preparation was performed according to the protocol “Ligation sequencing amplicons - PCR barcoding (SQK-LSK110 with EXP-PBC096)” by ONT.

The degenerate bases in the primer sequences (indicated in bold) follow the International Union of Biochemistry (IUB) nomenclature. The 27F-I primer set resulted in three sequence variants, while the 27F-II set generated 18 variants (16 forward, 2 reverse). A complete list of sequence variants is provided in **Supplementary Table 1**.

The barcoded libraries (27F-I and 27F-II) were loaded onto separate flow cells (FLO-MIN106D, R9.4.1, ONT) and sequenced independently using the MinION Mk1C device (ONT). Data acquisition was performed using MinKNOW (version 22.03.4, ONT) and Guppy 6.0.7. Both libraries were generated from DNA extracted using the same method.

### Bioinformatics processing and analysis

Raw sequencing data generated from full-length 16S rRNA gene amplicon sequencing using the two different primer sets on the ONT MinION platform were processed using EPI2ME (Oxford Nanopore Technologies) for taxonomic classification. The following workflow was applied to ensure high-quality data processing and accurate taxonomic assignment. Raw sequencing data were basecalled and demultiplexed using Guppy (version 6.5.7, Oxford Nanopore Technologies) in high-accuracy mode. Barcode demultiplexing was performed within Guppy using default settings. Reads with a quality score below 9 or truncated reads were excluded during this step. The resulting high-quality reads were subsequently processed using the Epi2me-Labs workflow (wf-16S) for taxonomic classification (GitHub wf-16s). This workflow includes primer and adapter trimming, length filtering, clustering of full-length 16S rRNA reads, and alignment against curated reference databases to enable taxonomic classification at the genus or species level. To validate and refine taxonomic assignments, the filtered reads were additionally aligned and oriented using Minimap2 (version 2.28), and full-length 16S sequences were extracted. Final classification was performed using the NCBI 16S rRNA reference database (ncbi_16s_18sRNA, January 2024 release). The classified reads were used to generate microbial community profiles, and relative abundances of bacterial taxa were calculated. To account for differences in sequencing depth across samples, normalization was applied using relative abundance measures. Further alpha diversity metrics and beta diversity analyses were computed to evaluate intra- and inter-sample diversity.

### Downstream statistical analysis

All statistical analyses and visualizations were conducted using the statistical programming language R, incorporating the *microeco* package (Liu et al., 2020). To compare the taxonomic composition at the genus level between datasets generated with the two primer sets (27F-I and 27F-II), Pearson’s correlation test was applied to relative abundance data. Further statistical comparisons, including relative abundance across all taxonomic levels and alpha diversity assessments via the Shannon Index, were performed using Wilcoxon signed-rank tests. Resulting p-values were adjusted using the Benjamini-Hochberg method to account for multiple comparisons. All tests considered the paired nature of the data, with a two-tailed p-value <0.05 deemed statistically significant.

## Results

Utilizing full-length 16S rRNA gene amplicon sequencing on the nanopore platform, we evaluated the efficiency of two primer sets: the standard 27F primer (designated as 27F-I) from ONT’s 16S Barcoding Kit (SQK-16S024) and a more degenerate variant (designated as 27F-II), designed to account for polymorphisms in conserved regions of the 16S rRNA gene. This comparison was conducted in the context of highly diverse bacterial communities from 80 human oropharyngeal swab samples. Demographic and baseline characteristics of the study cohort are summarized in **Supplementary Table S2**. The comparative primer strategy employed follows the four-primer PCR method outlined by Matsuo et al (Matsuo et al., 2021). This approach involves an initial PCR step utilizing a more degenerate 27F and 1492R primer pair [S-D-Bact-0008-c-S-20 and S-D-Bact-1492-a-A-22 (Klindworth et al., 2013)], followed by a barcoding PCR. Reads were aligned directly to the NCBI 16S database for taxonomic classification.

**Figure 1:**
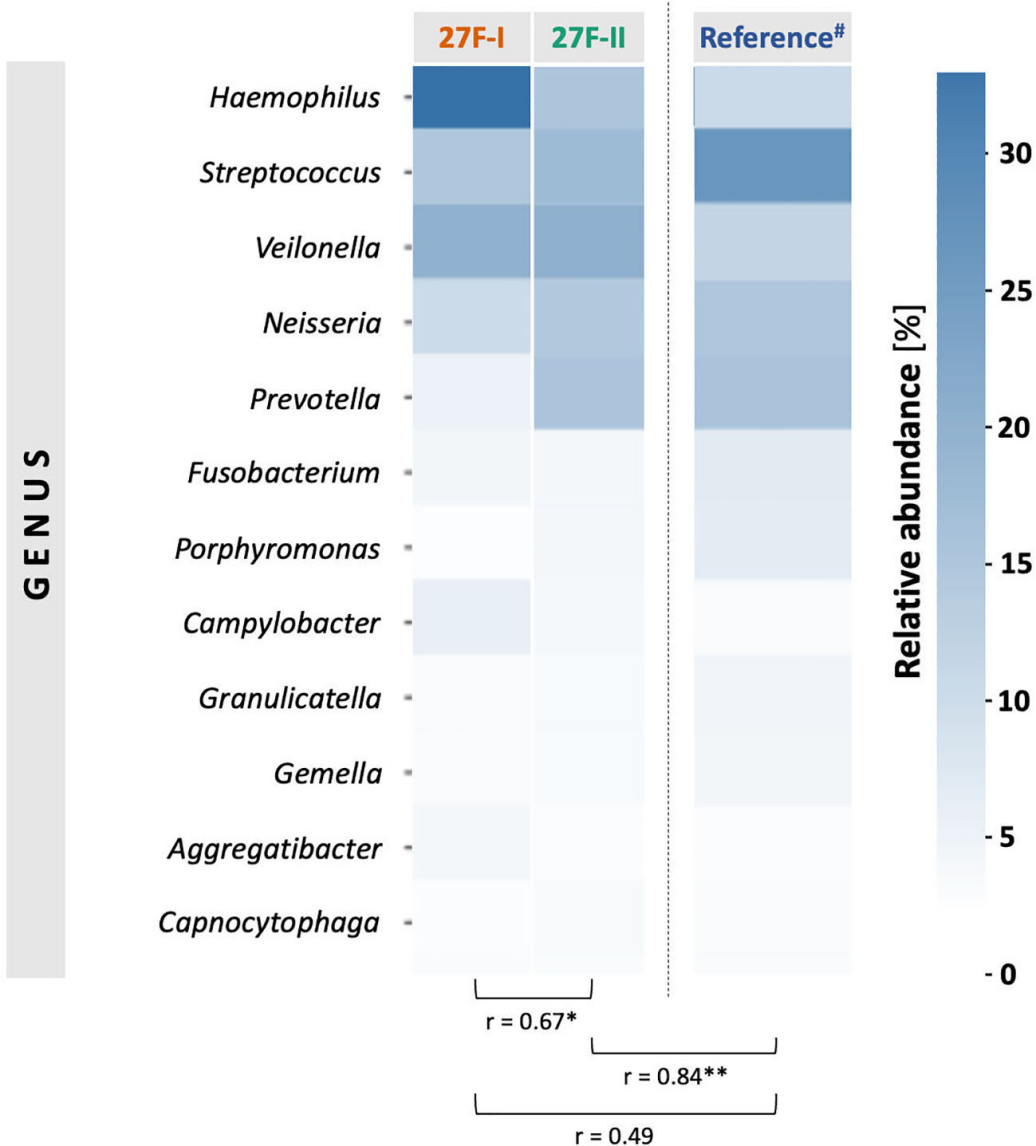
Comparison of the mean values of the relative genus abundance for the 12 most common taxa in the samples between the two primer sets using a heat map and the Pearson correlation (r). # Reference dataset from Ruan et al. with saliva samples from 1,989 healthy volunteers (Ruan et al., 2022). * - p-value <0.05, ** <p-value <0.01.

To globally compare the taxonomic profiles of the human oropharyngeal microbiota obtained with the two primer sets, the Pearson correlation coefficient (r) was calculated based on the average relative abundances of bacterial genera across all samples for each primer approach. The analysis showed only a moderate but statistically significant correlation (r = 0.67, p = 0.005) between the genera identified by the respective primer sets. To assess which primer more accurately represents the oropharyngeal microbiome, the taxonomic data generated using the 27F-I and 27F-II primers were compared to a reference dataset assembled by Ruan et al., which includes saliva samples from 1,989 healthy subjects (Ruan et al., 2022). The analysis revealed a strong and statistically significant correlation between the taxonomic profile of oral samples obtained with the 27F-II primer and the cited reference dataset (r = 0.86, p < 0.0001). In contrast, the correlation between the taxonomic profiles generated using the 27F-I primer and the reference dataset was weak and not statistically significant (r = 0.49, p = 0.06). **Figure 1** presents a heatmap comparing the relative abundance of the 12 most prevalent genera identified by the two primer sets.

**Figure 2:**
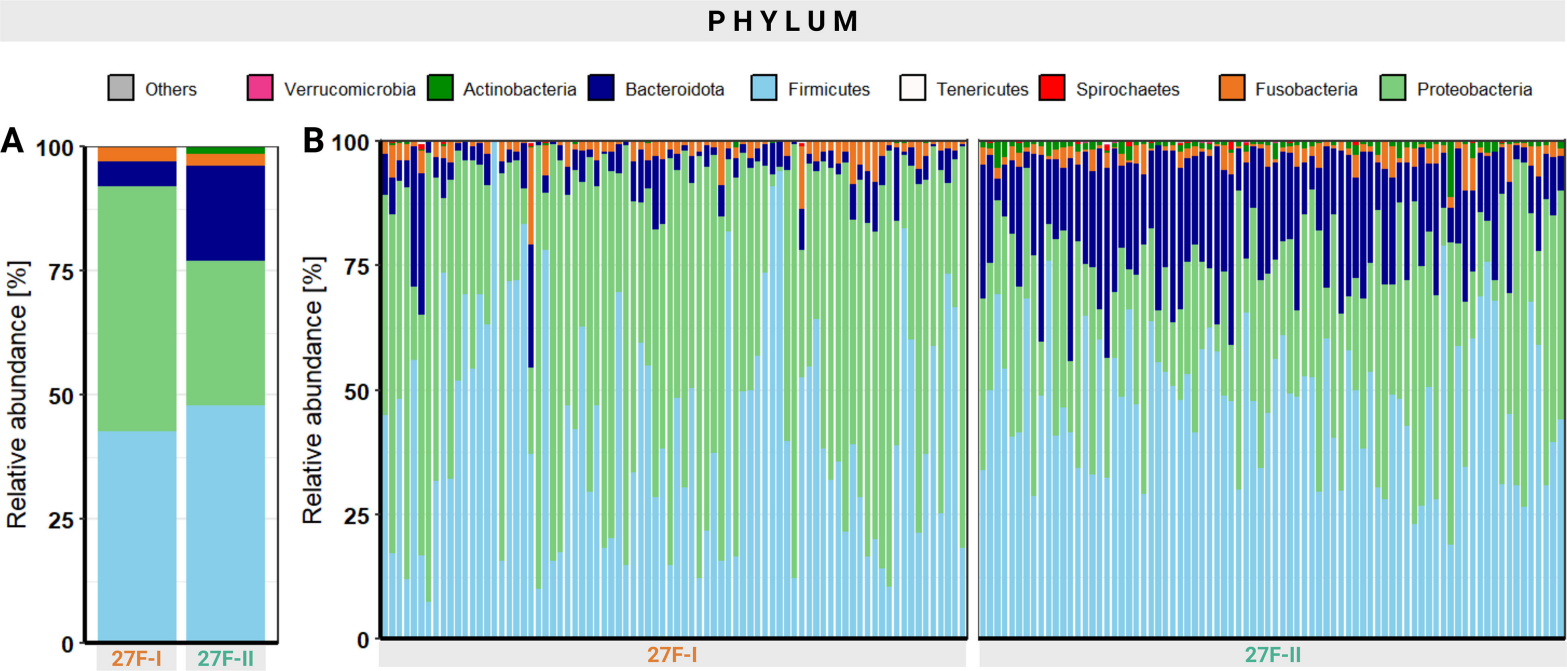
Overview of the relative abundance of the different phyla averaged over all samples **(A)** and at the individual sample level **(B)**.

A noticeable discrepancy in relative abundance is evident even at the phyla level. Across all analyzed samples, the 27F-I primer yielded a significantly higher proportion of Proteobacteria (49.2% vs. 29.2%, p < 0.001) and lower abundances of Bacteroidota (5.1% vs. 19.2%, p < 0.001), Actinobacteria (0.1% vs. 1.3%, p < 0.001), and Verrucomicrobia (0.001% vs. 0.08%, p < 0.001) compared to the 27F-II primer. **Figure 2** presents an overview of the relative abundance of different phyla, both as an average across all samples and at the individual sample level. Detailed quantitative data for all bacterial phyla are available in **Supplementary Table 3**.

**Figure 3:**
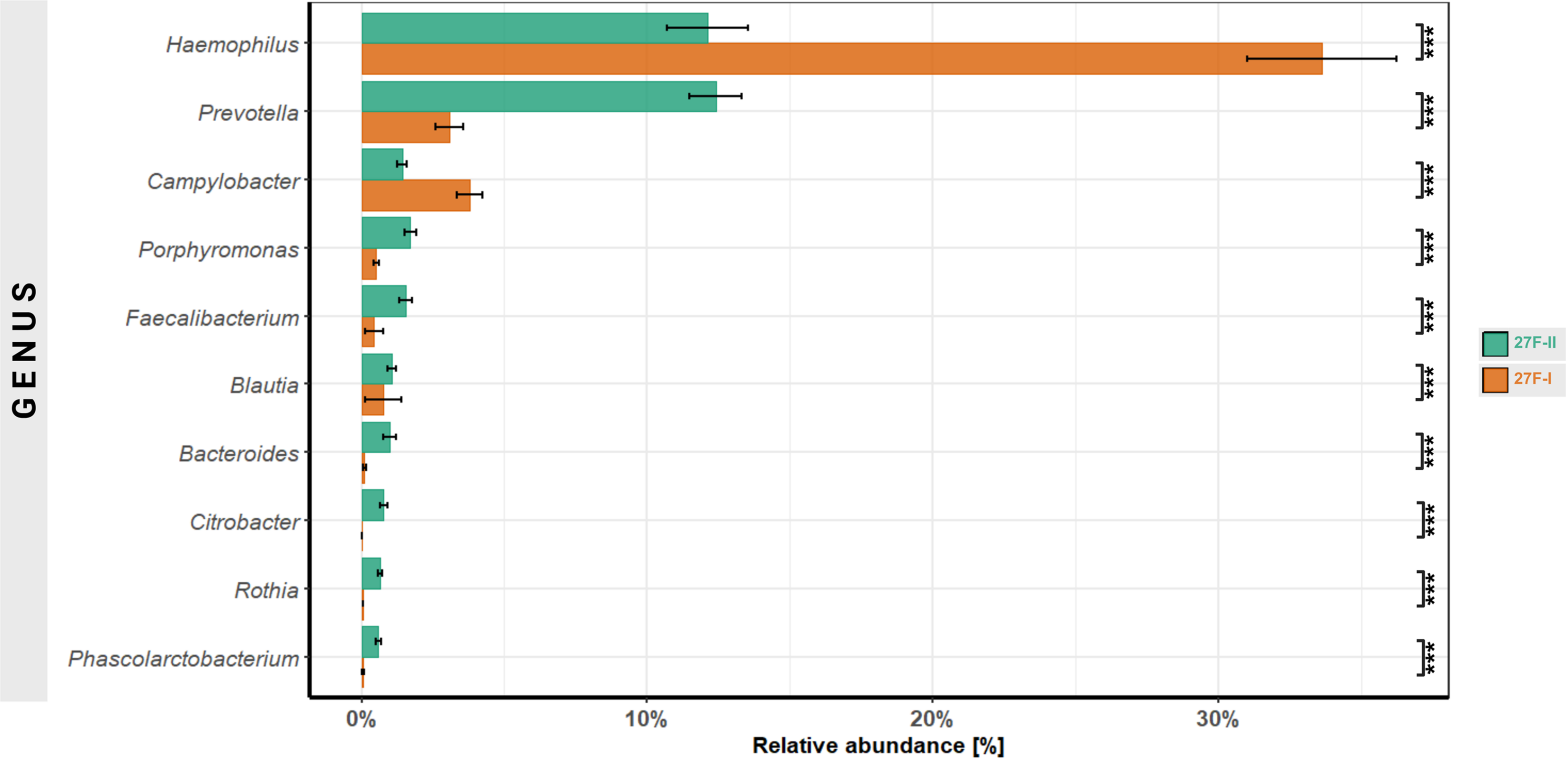
Comparison of genera with the most significant differences in abundance between the two primer approaches. ***–p-value < 0.001.

At the genus level, substantial differences in relative abundance were observed for 125 genera. Focusing on the 10 genera with the most significant differences, the 27F-I primer led to a higher relative abundance of Haemophilus (33.6% vs. 12.1%, p < 0.001) and Campylobacter (3.8% vs. 1.4%, p < 0.001). In contrast, the 27F-II primer detected significantly higher levels of Prevotella (3.1% vs. 12.4%, p < 0.001), Porphyromonas (0.5% vs. 1.7%, p < 0.001), Faecalibacterium (0.4% vs. 1.5%, p < 0.001), Blautia (0.076% vs. 1.07%, p < 0.001), Bacteroides (0.009% vs. 0.1%, p < 0.001), Citrobacter (0.00003% vs. 0.07%, p < 0.001), Rothia (0.004% vs. 0.064%, p < 0.001) and Phascolarctobacterium (0.005% vs. 0.0058%, p < 0.001) compared to 27F-I (**Figure 3**). Comprehensive quantitative data for all genera are provided in **Supplementary Table 4**. Since the 16S Barcoding Kit (SQK-16S024) containing 27F-I is validated only for genus-level resolution, species-level classification was not conducted.

**Figure 4:**
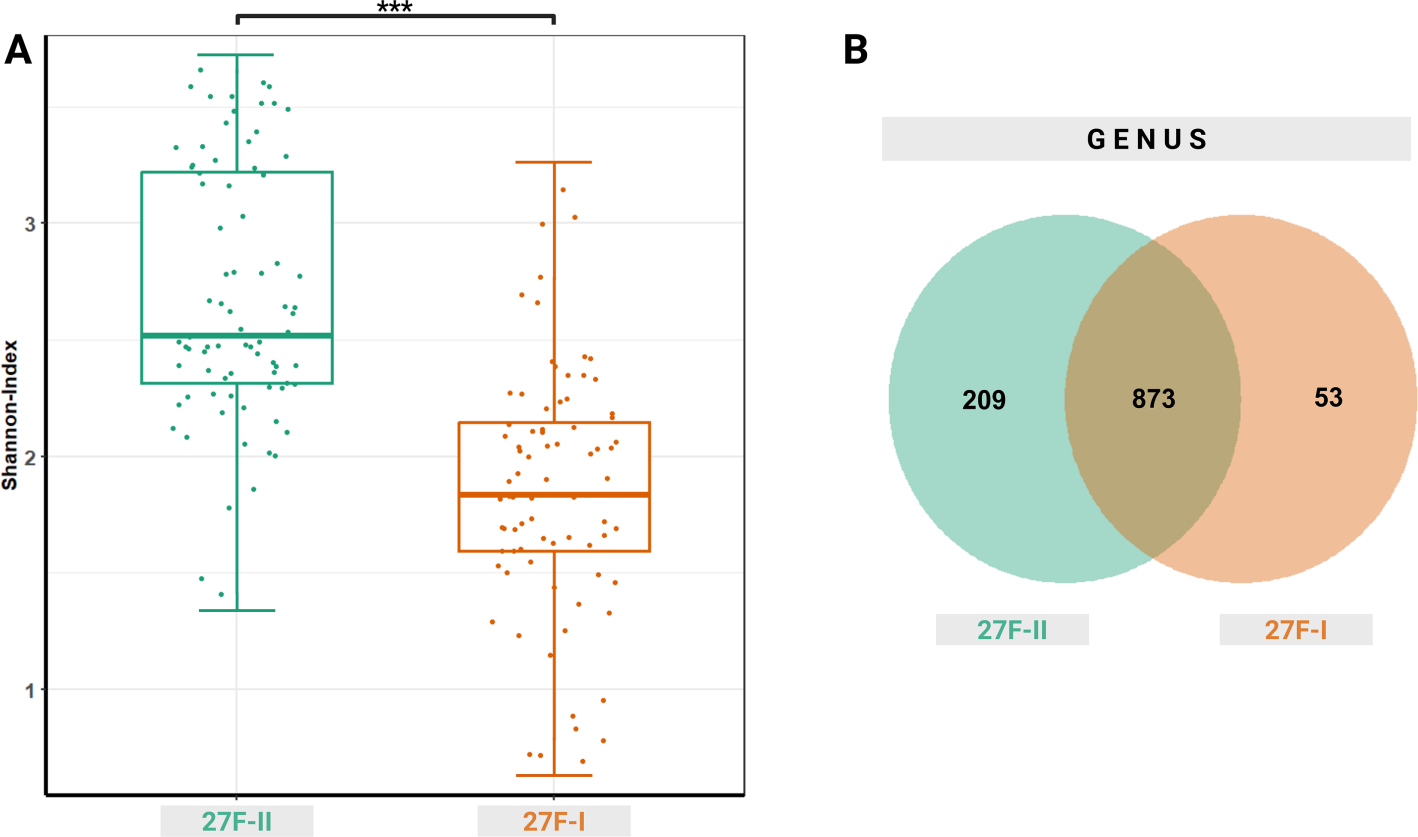
Alpha diversity represented as Shannon index **(A)** for the two primer approaches and a Venn diagram **(B)** showing the common and specifictaxonomic units at the genus level between the two primer sets used. ***– p-value < 0.001.

Beyond these taxonomic differences in the oropharyngeal microbiome, the choice of primer set also significantly influenced taxonomic diversity. The 27F-I primer detected fewer distinct amplicon sequence variants (ASV) in oropharyngeal swabs than the 27F-II primer, as reflected by a significantly lower Shannon index (1.850 vs. 2.684, p < 0.001), indicating reduced alpha diversity (**Figure 4**).

## Discussion

The advent of next-generation sequencing has transformed microbiology research, significantly enhancing our understanding of complex human gut bacterial communities. Among these technologies, nanopore sequencing has gained prominence due to its unique combination of cost efficiency, ease of use, high throughput, and superior taxonomic resolution, enabled by its ability to sequence long amplicons. Recent breakthroughs in sequencing accuracy have largely addressed one of the technology’s key limitations, marking a major milestone in the rapid evolution of the nanopore platform. These improvements have allowed nanopore sequencing to rival and, in some cases, surpass the capabilities of traditional short-read sequencing approaches. Additionally, the widely adopted 16S Barcoding Kit (SQK-16S024) from Oxford Nanopore Technologies (ONT) has further streamlined 16S rRNA gene sequencing, making it an accessible, fast, and cost-effective solution for microbiome research (Santos et al., 2020).

This study provides a systematic comparison of two primer sets with different levels of degeneracy for full-length 16S rRNA gene amplification from human oropharyngeal swab samples using nanopore sequencing. By adapting the approach of our previously published fecal microbiome study (Waechter et al., 2023) to the oral cavity, we extend the evidence that primer selection is a crucial determinant of sequencing outcome and diversity metrics in complex microbial environments.

The results demonstrate that the more degenerate primer set (27F-II) outperforms the standard ONT kit primer (27F-I) in capturing microbial diversity and in achieving a taxonomic composition more consistent with a reference dataset from nearly 2,000 healthy individuals’ salivary microbiota (Ruan et al., 2022). This is in line with previous findings from fecal samples, where the degenerate primer set led to higher biodiversity and a more balanced representation of key phyla, including Bacteroidota and Actinobacteria (Waechter et al., 2023). Our current data confirm this pattern in the oropharyngeal microbiome, suggesting that primer-induced amplification bias is not limited to gut environments but similarly affects oral microbial profiling.

The discrepancies observed between the two primer sets are particularly pronounced at both the phylum and genus levels. The 27F-I primer set yielded a microbial profile dominated by Proteobacteria and Haemophilus, reflecting overamplification of specific taxa. In contrast, the degenerate primer set enabled a more diverse detection spectrum, revealing increased levels of clinically relevant genera such as Prevotella, Porphyromonas, and Faecalibacterium, which are often underrepresented in datasets generated with less degenerate primers. These findings highlight the risk of skewed taxonomic inference when using primers with limited degeneracy, especially in environments with high microbial variability such as the oropharyngeal cavity.

Importantly, the more degenerate primer set also led to significantly higher alpha diversity, as indicated by the Shannon index. This reinforces the notion that primer degeneracy enhances the detection of low-abundance taxa, contributing to a more comprehensive and ecologically valid microbiome profile. The correlation with the large-scale salivary microbiome dataset by Ruan et al. further strengthens the validity of the degenerate primer set for oral microbial community profiling and supports its use as a methodological standard in future studies (Klindworth et al., 2013).

Our findings also have important implications beyond primer performance. While full-length 16S rRNA gene sequencing using long-read platforms such as ONT allows comprehensive coverage across all nine hypervariable regions (V1–V9), this does not inherently guarantee higher taxonomic resolution for all bacterial clades. Several studies have demonstrated that targeted short-read sequencing, particularly of the V1 - V4 or V3 - V4 regions using Illumina technology, can outperform full-length approaches in certain contexts. This is especially true for taxa whose discriminative nucleotide signatures are concentrated in specific regions of the gene, such as *Bifidobacterium*, *Lactobacillus*, or *Enterobacteriacea* members, where short-read methods have shown better genus- or even species-level concordance with whole-genome data (Janssen et al., 2018; Macip et al., 2025).

Moreover, the error profile of long-read sequencing, although significantly improved in recent ONT chemistry (e.g., Q20+ kits), may still impair taxonomic resolution at lower ranks when not properly corrected (Liu-Wei et al., 2024). This is particularly relevant in clinical diagnostics, where misclassification of near-neighbor taxa may lead to false-positive or false-negative results (Gu et al., 2018). As such, the decision between short- and long-read platforms should be guided by the biological context, the expected diversity and complexity of the sample type, and the resolution required for the intended application. For instance, high-throughput surveillance studies may prioritize cost-effective short-read platforms with robust pipelines, whereas exploratory profiling of under-characterized niches may benefit from the broader coverage of full-length sequencing (Wenger et al., 2019). Clinical applications may require additional benchmarking or validation with mock communities to ensure sufficient taxonomic precision and reproducibility.

### Limitations

This study has several limitations that merit discussion. The most important constraint is the absence of an internal benchmarking strategy for evaluating the taxonomic fidelity of the two primer sets. Unlike previous studies that incorporated mock communities, well-characterized reference strains or internal benchmark analyses with short-read sequencing platforms to assess sequencing accuracy and amplification bias (Hugerth and Andersson, 2017), our analysis relied on an indirect benchmarking approach: we compared our sequencing results with a large-scale reference dataset from healthy individuals’ saliva microbiota published by Ruan et al (Ruan et al., 2022). While this comparison provides a useful external anchor point, it entails several methodological caveats First, the reference data were generated using short-read sequencing targeting the V3 - V4 or V4 hypervariable regions of the 16S rRNA gene, which contrasts with our approach of full-length 16S rRNA gene sequencing on the ONT platform, along with all the implications discussed earlier. Second, the two studies used different taxonomic classification frameworks: the Ruan dataset was annotated using the SILVA database, whereas our analysis was based on the NCBI 16S rRNA reference database due to its native integration into the Epi2me workflow. These differences in region selection, sequencing platform, and taxonomic backbone likely contribute to discrepancies in observed microbial profiles and complicate direct comparison. They also highlight the broader challenge of standardization in microbiome research, particularly when studies aim to benchmark across heterogeneous analytical pipelines.

Third, the DNA extraction methods used in the reference study may differ from our protocol. DNA isolation procedures have a well-documented impact on microbial community composition, especially when comparing mechanical lysis (e.g., bead-beating) with enzymatic or chemical methods (Yuan et al., 2012; Costea et al., 2017).

Fourth, the human donors in the reference study are likely to differ from our cohort in lifestyle, diet, geography, and even oral hygiene practices - all of which are known to significantly influence the oral microbiome (Ding and Schloss, 2014). Although we controlled for acute inflammatory conditions and standardized sample collection, we cannot rule out the influence of cohort-specific variables that might confound direct comparisons.

Despite these limitations, we argue that the comparison with the large-scale reference dataset provides a reasonable orientation for assessing the relative performance of the two primer sets. In the absence of a universally accepted gold standard for oral 16S rRNA sequencing, particularly one using full-length amplicons, such external benchmarks remain a pragmatic alternative. Moreover, the broader question remains whether a “true” benchmark for microbiome profiling can exist at all, given the multiplicity of sequencing platforms, primer sets, and bioinformatic pipelines in current use. Therefore, our findings should be interpreted as contextually robust rather than absolutely definitive.

## Conclusion and future directions

Recent improvements in ONT sequencing chemistry and basecalling have substantially increased the accuracy of full-length 16S rRNA gene sequencing, thereby enhancing its potential to resolve complex microbial communities with higher taxonomic resolution than conventional short-read approaches. Given its cost-efficiency, scalability, and ability to sequence full-length amplicons in real time, the ONT platform is poised to gain increasing importance in oral and oropharyngeal microbiome research.

Our study presents a comparative analysis of two primer sets with different levels of degeneracy for nanopore-based 16S rRNA gene sequencing of human oropharyngeal swabs. We demonstrate that the widely used standard 27F primer (27F-I) introduces measurable amplification bias, whereas a more degenerate variant (27F-II) yields richer and more representative taxonomic profiles. These findings underscore the critical role of primer selection in shaping microbiome readouts and support the broader use of degenerate primers for accurate and unbiased profiling in complex oral environments.

Looking ahead, future studies should aim for greater methodological harmonization, particularly in the design and selection of primer sets. The current lack of interoperability among primer strategies remains a major obstacle to reproducibility and cross-study comparability. Establishing community-wide standards for primer choice, as well as unified guidelines for the selection of taxonomic reference databases across anatomical niches and sequencing platforms, will be essential for advancing microbiome research toward clinical and translational applications.

## Data Availability

The original contributions presented in the study are included in the article/**Supplementary Material**, further inquiries can be directed to the corresponding author/s.

## References

[B1] Aggarwal N. Kitano S. Puah G. R. Y. Kittelmann S. Hwang I. Y. Chang M. W. (2023). Microbiome and human health: current understanding, engineering, and enabling technologies. Chem. Rev. 123, 31–72. doi: 10.1021/acs.chemrev.2c00431, PMID: 36317983 PMC9837825

[B2] GitHub wf-16s. Available online at: https://github.com/epi2me-labs/wf-16s (Accessed March 12, 2025).

[B3] Bao L. Zhang C. Dong J. Zhao L. Li Y. Sun J. (2020). Oral microbiome and SARS-coV-2: beware of lung co-infection. Front. Microbiol. 11. doi: 10.3389/fmicb.2020.01840, PMID: 32849438 PMC7411080

[B4] Burcham Z. M. Garneau N. L. Comstock S. S. Tucker R. M. Knight R. Metcalf J. L. . (2020). Patterns of oral microbiota diversity in adults and children: A crowdsourced population study. Sci. Rep. 10, 2133. doi: 10.1038/s41598-020-59016-0, PMID: 32034250 PMC7005749

[B5] Charlson E. S. Bittinger K. Haas A. R. Fitzgerald A. S. Frank I. Yadav A. . (2011). Topographical continuity of bacterial populations in the healthy human respiratory tract. Am. J. Respir. Crit. Care Med. 184, 957–963. doi: 10.1164/rccm.201104-0655oc, PMID: 21680950 PMC3208663

[B6] Costea P. I. Zeller G. Sunagawa S. Pelletier E. Alberti A. Levenez F. . (2017). Towards standards for human fecal sample processing in metagenomic studies. Nat. Biotechnol. 35, 1069–1076. doi: 10.1038/nbt.3960, PMID: 28967887

[B7] Deissová T. Zapletalová M. Kunovský L. Kroupa R. Grolich T. Kala Z. . (2023). 16S rRNA gene primer choice impacts off-target amplification in human gastrointestinal tract biopsies and microbiome profiling. Sci. Rep. 13, 12577. doi: 10.1038/s41598-023-39575-8, PMID: 37537336 PMC10400661

[B8] Ding T. Schloss P. D. (2014). Dynamics and associations of microbial community types across the human body. Nature 509, 357–360. doi: 10.1038/nature13178, PMID: 24739969 PMC4139711

[B9] Frank J. A. Reich C. I. Sharma S. Weisbaum J. S. Wilson B. A. Olsen G. J. (2008). Critical evaluation of two primers commonly used for amplification of bacterial 16S rRNA genes. Appl. Environ. Microb. 74, 2461–2470. doi: 10.1128/aem.02272-07, PMID: 18296538 PMC2293150

[B10] Gu W. Miller S. Chiu C. Y. (2018). Clinical metagenomic next-generation sequencing for pathogen detection. Annu. Rev. Pathol. Mech. Dis. 14, 1–20. doi: 10.1146/annurev-pathmechdis-012418-012751, PMID: 30355154 PMC6345613

[B11] Hugerth L. W. Andersson A. F. (2017). Analysing microbial community composition through amplicon sequencing: from sampling to hypothesis testing. Front. Microbiol. 8. doi: 10.3389/fmicb.2017.01561, PMID: 28928718 PMC5591341

[B12] Huttenhower C. Gevers D. Knight R. Abubucker S. Badger J. H. Chinwalla A. T. . (2012). Structure, function and diversity of the healthy human microbiome. Nature 486, 207–214. doi: 10.1038/nature11234, PMID: 22699609 PMC3564958

[B13] Janssen S. McDonald D. Gonzalez A. Navas-Molina J. A. Jiang L. Xu Z. Z. . (2018). Phylogenetic placement of exact amplicon sequences improves associations with clinical information. mSystems 3. doi: 10.1128/msystems.00021-18, PMID: 29719869 PMC5904434

[B14] Kageyama S. Takeshita T. (2024). Development and establishment of oral microbiota in early life. J. Oral. Biosci. 66, 300–303. doi: 10.1016/j.job.2024.05.001, PMID: 38703995

[B15] Kim B. Y. Gellert H. R. Church S. H. Suvorov A. Anderson S. S. Barmina O. . (2024). Single-fly genome assemblies fill major phylogenomic gaps across the drosophilidae tree of life. PloS Biol. 22, e3002697. doi: 10.1371/journal.pbio.3002697, PMID: 39024225 PMC11257246

[B16] Klindworth A. Pruesse E. Schweer T. Peplies J. Quast C. Horn M. . (2013). Evaluation of general 16S ribosomal RNA gene PCR primers for classical and next-generation sequencing-based diversity studies. Nucleic Acids Res. 41, e1–e1. doi: 10.1093/nar/gks808, PMID: 22933715 PMC3592464

[B17] Lee Y.-H. Chung S. W. Auh Q.-S. Hong S.-J. Lee Y.-A. Jung J. . (2021). Progress in oral microbiome related to oral and systemic diseases: an update. Diagnostics 11, 1283. doi: 10.3390/diagnostics11071283, PMID: 34359364 PMC8306157

[B18] Lemon K. P. Klepac-Ceraj V. Schiffer H. K. Brodie E. L. Lynch S. V. Kolter R. (2010). Comparative analyses of the bacterial microbiota of the human nostril and oropharynx. mBio 1. doi: 10.1128/mbio.00129-10, PMID: 20802827 PMC2925076

[B19] Liu C. Cui Y. Li X. Yao M. (2020). Microeco: an R package for data mining in microbial community ecology. FEMS Microbiol. Ecol. 97. doi: 10.1093/femsec/fiaa255, PMID: 33332530

[B20] Liu-Wei W. van der Toorn W. Bohn P. Hölzer M. Smyth R. P. von Kleist M. (2024). Sequencing accuracy and systematic errors of nanopore direct RNA sequencing. BMC Genom 25, 528. doi: 10.1186/s12864-024-10440-w, PMID: 38807060 PMC11134706

[B21] Macip G. Soler-Comas A. Palomeque A. Motos A. Llonch B. Canseco-Ribas J. . (2025). Comparative analysis of illumina and oxford nanopore sequencing platforms for 16S rRNA profiling of respiratory microbial communities. Sci. Rep. 15, 33688. doi: 10.1038/s41598-025-18768-3, PMID: 41023034 PMC12480654

[B22] Malla M. A. Dubey A. Kumar A. Yadav S. Hashem A. Abd_Allah E. F. (2019). Exploring the human microbiome: the potential future role of next-generation sequencing in disease diagnosis and treatment. Front. Immunol. 9. doi: 10.3389/fimmu.2018.02868, PMID: 30666248 PMC6330296

[B23] Matsuo Y. Komiya S. Yasumizu Y. Yasuoka Y. Mizushima K. Takagi T. . (2021). Full-length 16S rRNA gene amplicon analysis of human gut microbiota using minION^TM^ nanopore sequencing confers species-level resolution. BMC Microbiol. 21, 35. doi: 10.1186/s12866-021-02094-5, PMID: 33499799 PMC7836573

[B24] McDonalda D. Hyde E. Debelius J. W. Morton J. T. Gonzalez A. Ackermann G. . (2018). American gut: an open platform for citizen-science microbiome research. Biorxiv. 3, 277970. doi: 10.1101/277970, PMID: 29795809 PMC5954204

[B25] Peng X. Cheng L. You Y. Tang C. Ren B. Li Y. . (2022). Oral microbiota in human systematic diseases. Int. J. Oral. Sci. 14, 14. doi: 10.1038/s41368-022-00163-7, PMID: 35236828 PMC8891310

[B26] Piters W. A. A. Binkowska J. Bogaert D. (2020). Early life microbiota and respiratory tract infections. Cell Host Microbe 28, 223–232. doi: 10.1016/j.chom.2020.07.004, PMID: 32791114

[B27] Ravi R. K. Walton K. Khosroheidari M. (2018). Disease gene identification, methods and protocols. Methods Mol. Biol. 1706, 223–232. doi: 10.1007/978-1-4939-7471-9_12, PMID: 29423801

[B28] Ruan X. Luo J. Zhang P. Howell K. (2022). The Salivary Microbiome Shows a High Prevalence of Core Bacterial Members yet Variability across Human Populations. NPJ Biofilms Microbiomes 8, 85. doi: 10.1038/s41522-022-00343-7, PMID: 36266278 PMC9584946

[B29] Sampaio-Maia B. Monteiro-Silva F. (2014). Acquisition and maturation of oral microbiome throughout childhood: an update. Dent. Res. J. 11, 291–301., PMID: 25097637 PMC4119360

[B30] Santos A. Aerle R. barrientos L. Martinez-Urtaza J. (2020). Computational methods for 16S metabarcoding studies using nanopore sequencing data. Comput. Struct. Biotechnol. J. 18, 296–305. doi: 10.1016/j.csbj.2020.01.005, PMID: 32071706 PMC7013242

[B31] Waechter C. Fehse L. Welzel M. Heider D. Babalija L. Cheko J. . (2023). Comparative analysis of full-length 16s ribosomal RNA genome sequencing in human fecal samples using primer sets with different degrees of degeneracy. Front. Genet. 14. doi: 10.3389/fgene.2023.1213829, PMID: 37564874 PMC10411958

[B32] Wenger A. M. Peluso P. Rowell W. J. Chang P.-C. Hall R. J. Concepcion G. T. . (2019). Accurate circular consensus long-read sequencing improves variant detection and assembly of a human genome. Nat. Biotechnol. 37, 1155–1162. doi: 10.1038/s41587-019-0217-9, PMID: 31406327 PMC6776680

[B33] Yuan S. Cohen D. B. Ravel J. Abdo Z. Forney L. J. (2012). Evaluation of methods for the extraction and purification of DNA from the human microbiome. PloS One 7, e33865. doi: 10.1371/journal.pone.0033865, PMID: 22457796 PMC3311548

