# Supplementary material for: Comparative analysis of full-length 16s ribosomal RNA gene sequencing in human oropharyngeal swabs using primer sets with different degrees of degeneracy
Source: Front Cell Infect Microbiol. 2025 Nov 17;15:1658615. doi: 10.3389/fcimb.2025.1658615 (PMC12665744; doi:10.3389/fcimb.2025.1658615)
Supplement: Supplementary file 1 [file DataSheet1.pdf]

Supplementary Table 2 – Baseline Characteristics

	Baseline Characteristics (n= 80)
**Age***	63 years (12,2 years)
**Male Sex**	68% (54/80)
**Body Mass Index***	29,7 kg/m^2 (5,8 kg/m^2)
**Caucasian Ethnicity**	96% (77/80)
**Active Smoking**	8% (6/80)
**Use of Mouthwash**	35% (28/80)
**Paradontal Disease**	34% (27/80)
**Full dentures**	11% (9/80)
**C-reactive Protein***	4,2 mg/L (4,5 mg/L)
**Leukocyte count***	5,7 G/L (0,1 G/L)

* data represents mean and standard deviation
